# Design of predictive model to optimize the solubility of Oxaprozin as nonsteroidal anti-inflammatory drug

**DOI:** 10.1038/s41598-022-17350-5

**Published:** 2022-07-30

**Authors:** Sameer Alshehri, Mohammed Alqarni, Nader Ibrahim Namazi, Ibrahim A. Naguib, Kumar Venkatesan, Yasser O. Mosaad, Mahboubeh Pishnamazi, Amal M. Alsubaiyel, Mohammed A. S. Abourehab

**Affiliations:** 1 Department of Pharmaceutics and Industrial Pharmacy, College of Pharmacy, Taif University, P.O. Box 11099, Taif, 21944 Saudi Arabia; 2 Department of Pharmaceutical Chemistry, College of Pharmacy, Taif University, P. O. Box 11099, Taif, 21944 Saudi Arabia; 3 Pharmaceutics and Pharmaceutical Technology Department, College of Pharmacy, Taibah University, Al Madinah Al Munawarah, 30001 Saudi Arabia; 4 Department of Pharmaceutical Chemistry, College of Pharmacy, King Khalid University, Abha, 62529 Kingdom of Saudi Arabia; 5 Department of Pharmacy Practice and Clinical Pharmacy, Faculty Pharmacy, Future Unibversity in Egypt, New Cairo, 11835 Egypt; 6 Institute of Research and Development, Duy Tan University, Da Nang, 550000 Viet Nam; 7 The Faculty of Pharmacy, Duy Tan University, Da Nang, 550000 Viet Nam; 8 Department of Pharmaceutics, College of Pharmacy, Qassim University, Buraidah, 52571 Saudi Arabia; 9 Department of Pharmaceutics, Faculty of Pharmacy, Umm Al-Qura University, Makkah, 21955 Saudi Arabia; 10 Department of Pharmaceutics and Industrial Pharmacy, College of Pharmacy, Minia University, Minia, 61519 Egypt

**Keywords:** Chemistry, Green chemistry

## Abstract

These days, many efforts have been made to increase and develop the solubility and bioavailability of novel therapeutic medicines. One of the most believable approaches is the operation of supercritical carbon dioxide fluid (SC-CO_2_). This operation has been used as a unique method in pharmacology due to the brilliant positive points such as colorless nature, cost-effectives, and environmentally friendly. This research project is aimed to mathematically calculate the solubility of Oxaprozin in SC-CO_2_ through artificial intelligence. Oxaprozin is a nonsteroidal anti-inflammatory drug which is useful in arthritis disease to improve swelling and pain. Oxaprozin is a type of BCS class II (Biopharmaceutical Classification) drug with low solubility and bioavailability. Here in order to optimize and improve the solubility of Oxaprozin, three ensemble decision tree-based models including random forest (RF), Extremely random trees (ET), and gradient boosting (GB) are considered. 32 data vectors are used for this modeling, moreover, temperature and pressure as inputs, and drug solubility as output. Using the MSE metric, ET, RF, and GB illustrated error rates of 6.29E−09, 9.71E−09, and 3.78E−11. Then, using the R-squared metric, they demonstrated results including 0.999, 0.984, and 0.999, respectively. GB is selected as the best fitted model with the optimal values including 33.15 (K) for the temperature, 380.4 (bar) for the pressure and 0.001242 (mole fraction) as optimized value for the solubility.

## Introduction

**Figure 1:**
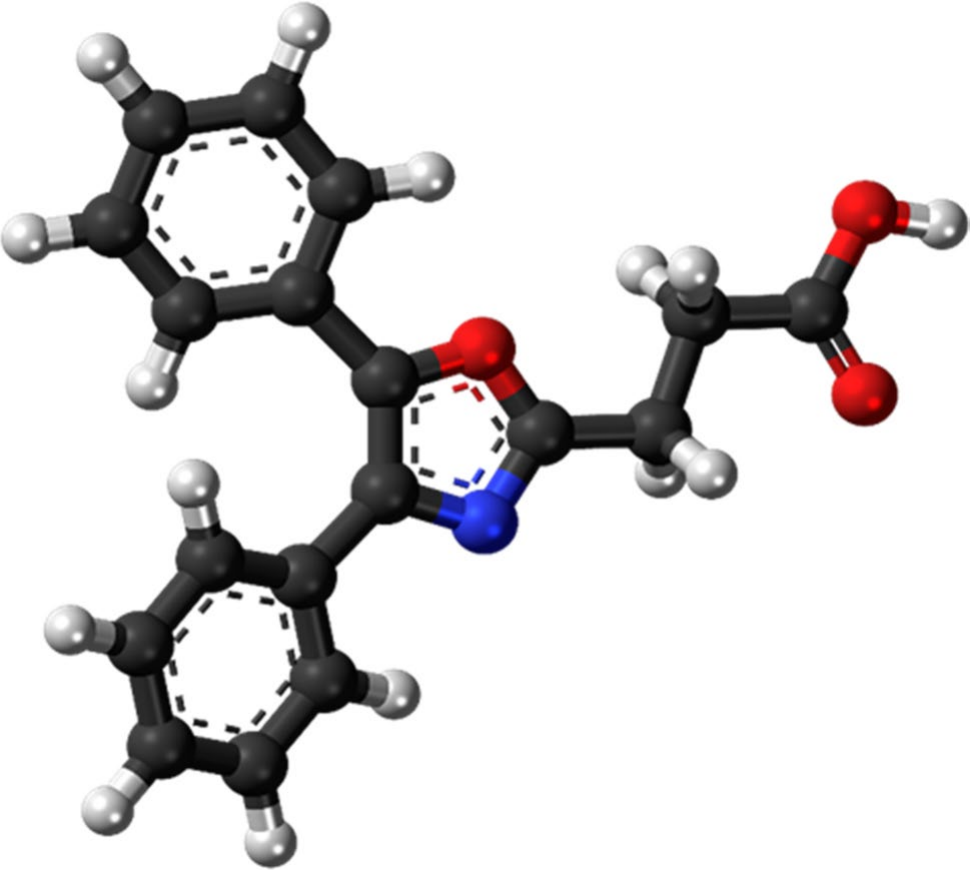
Schematic demonstration of Oxaprozin^10^.

Remarkable progression in pharmaceutical industry has paved the way towards creating novel therapeutic drugs for treating various challenging diseases^1,2^. Despite noteworthy development, poor solubility of active pharmaceutical ingredients (APIs) can be considered as the most prominent limitations for drug development^3,4^. Oxaprozin (C_18_H_15_NO_3_) can be recognized as one of the commonly-employed non-steroidal anti-inflammatory (NSAID) drug^5,6^. The analgesic and antipyretic characteristics of this propionic acid derivative has made it promising to appropriately alleviate the pain of acute/chronic disorders such as inflammation, swelling, osteoarthritis and rheumatoid arthritis^7,8^. Figure 1 presents the ball-stick demonstration of Oxaprozin. This NSAID drug possesses great ability to decline the formation of prostaglandin precursors from arachidonic acid via cyclo-oxygenase inhibition, which causes significant reduction in pain/inflammatory responses. Oxaprozin has shown superior efficacy compared to aspirin or piroxicam in the treatment of osteoarthritis^9^.

To improve the solubility of drugs, the indisputable role of solvents can’t be ignored. These days, supercritical fluids (SCFs) are known as an innovative technique that demonstrates their efficiency for particle formation. This novel approach can overcome some disadvantages of conventional technologies such as crushing, crystallization and precipitation^11,12^. Supercritical carbon dioxide (SC-CO_2_) is being frequently applied to fractionate the precious components in pharmaceutical processes due to possessing noteworthy properties such as abundancy, colorless nature, cost-effectiveness, and environmentally benign characteristic^13^. Due to the importance of solubility in SC-CO_2_ for the design and development of novel drugs, the conduction of experimental investigation for evaluating the solubility of these drugs is of great importance^14^. Despite the great importance for obtaining the solubility of drugs, the existence of some economic/operational problems such as difficulty in solute–solvent interactions in SC-CO_2_ system and high cost have limited the conduction of experimental investigation.

Therefore, development of mathematical modeling approaches to predict the solubility amount of disparate types of drugs can be an appropriate option to optimize the time and cost of processing. Nowadays, AI has been introduced as a promising predictive tool to measure the solubility of drugs, numerically. Apart from pharmacology, AI has found its indisputable role in disparate knowledge related to chemical engineering such as extraction, purification, separation, crystallization and chemical reactor engineering^15^. In most scientific fields, machine learning (ML) techniques are known as common computational procedures, including regression trees, neural networks, support vector machines. A variety of relationships between inputs and outputs are extracted by these models^16–18^.

The Decision Tree (DT) is one of the typically used learning models. A weak model is a simple predictor that is only likely to be better than a random estimator. The results of many base DT models are aggregated to form a stronger model in tree-based ensemble methods^19,20^.

Bagging and boosting are two of the most effective improvement strategies with Decision Trees. Bagging (Bootstrap Aggregating), developed by Breiman^21^, is one of the most basic and straightforward ensemble techniques, demonstrating outstanding performance while reducing variance and preventing overfitting. The Bagging algorithm is more diverse because of the bootstrap approach, which replicates and generates subsets of training data. All of the subsets are used to fit different basic estimators, and the final prediction results are compiled using a majority-vote method^21,22^.

One other ensemble method based on the Freund and Schapiro’s study is boosting^23^. The aim of this research was optimization of Oxaprozin solubility within supercritical fluid by applying different machine learning models to find the best model for that.

By progressively reweighting the training data, this approach differs from Bagging in that it generates a diverse set of basic learners. A higher weight will be given to each sample whose estimation was weaker than the previous estimator's in the subsequent training step. As a result, in subsequent bootstrap samples, it is more likely that training samples with weak estimates will appear, allowing bias to be effectively removed. Based on their prediction performance, the base estimators are weighted in the final Boosting algorithm model. A random forest model, Extra Trees, and Gradient Boosting model were all considered for inclusion in this research^24–27^.

## Experimental

Various predictive models in this research have been investigated and developed based on the experimental investigation of Khoshmaram et al. They experimentally measured the solubility of Oxaprozin using the combination of static and gravimetric techniques via a pressure–volume-temperature (PVT) cell^14^. This system can be filled with up to 0.4 L Oxaprozin and supercritical liquid. The adjustment of two momentous parameters for evaluating the solubility of drugs (temperature and pressure) in the PVT cell is an important advantage. In the PVT cell, increment of pressure causes the manufacturing of SC-CO_2_ in the liquefaction unit. Then, the condensed solvent moves through the inline filter with the aim of purifying the solvent. In the next step, purified solvent enters a surge tank before the PVT cell. The controlling process of SC-CO_2_ and Oxaprozin temperatures was implemented applying heating elements insulated by a PTFE layer.

## Data set

**Figure 2:**
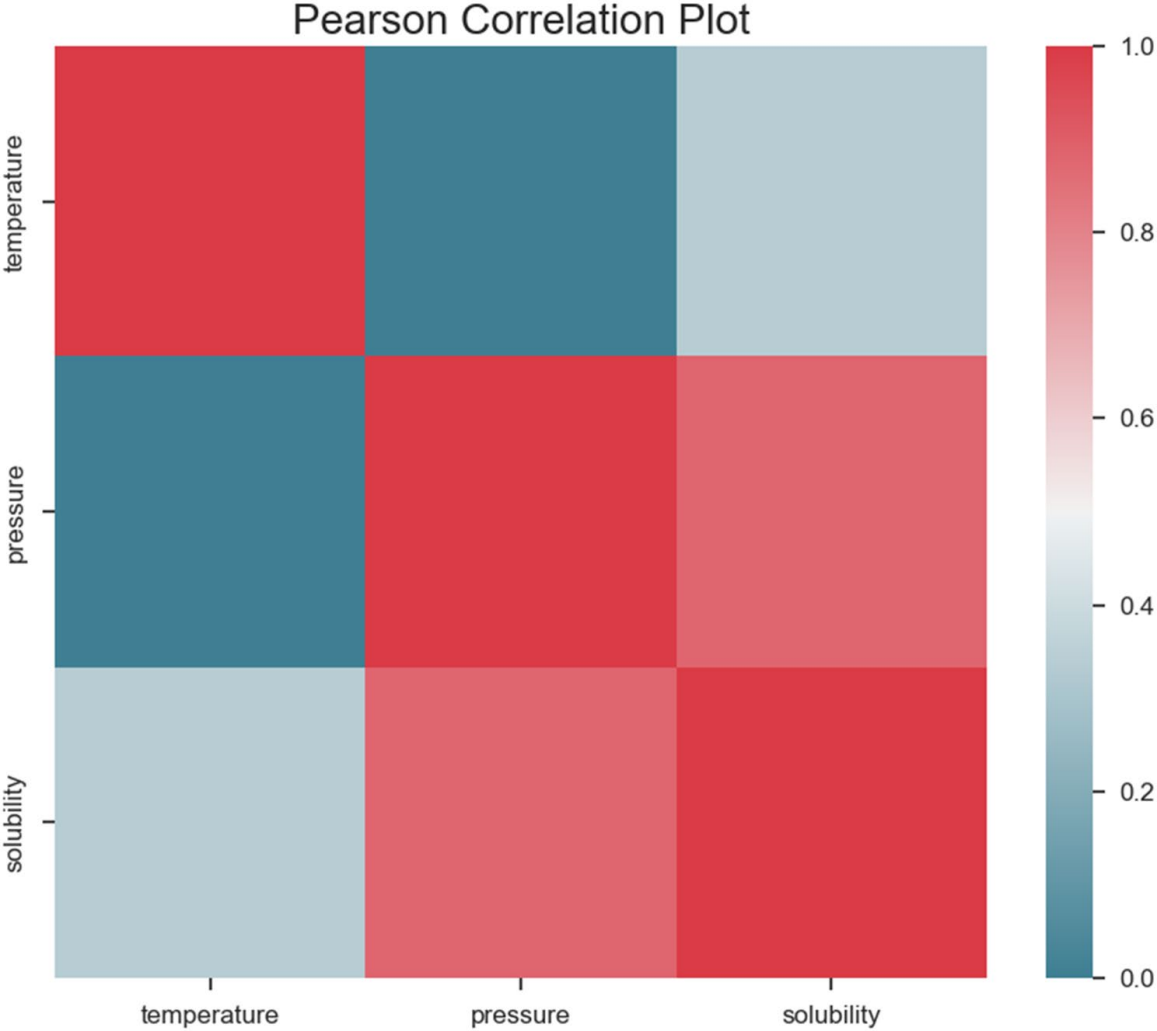
Pearson correlation plot.

This study's dataset is derived from^14^ that have just 32 data vectors. Each vector has two input parameters (pressure and temperature) and one output (solubility). The dataset is shown in Table 1 and Pearson correlation^28^ of parameters are shown in Fig. 2.

**Table 1:** The whole dataset: 32 data vectors, where each vector has two input parameters (pressure and temperature) and one output (solubility).

No	Temperature (K)	Pressure (bar)	Solubility (mole fraction)
1	3.08 × 10^2^	1.20 × 10^2^	8.19 × 10^–5^
2	3.08 × 10^2^	1.60 × 10^2^	1.58 × 10^–4^
3	3.08 × 10^2^	2.00 × 10^2^	2.24 × 10^–4^
4	3.08 × 10^2^	2.40 × 10^2^	2.80 × 10^–4^
5	3.08 × 10^2^	2.80 × 10^2^	3.44 × 10^–4^
6	3.08 × 10^2^	3.20 × 10^2^	4.06 × 10^–4^
7	3.08 × 10^2^	3.60 × 10^2^	4.73 × 10^–4^
8	3.08 × 10^2^	4.00 × 10^2^	5.33 × 10^–4^
9	3.18 × 10^2^	1.20 × 10^2^	7.55 × 10^–5^
10	3.18 × 10^2^	1.60 × 10^2^	1.41 × 10^–4^
11	3.18 × 10^2^	2.00 × 10^2^	2.45 × 10^–4^
12	3.18 × 10^2^	2.40 × 10^2^	3.56 × 10^–4^
13	3.18 × 10^2^	2.80 × 10^2^	4.64 × 10^–4^
14	3.18 × 10^2^	3.20 × 10^2^	5.58 × 10^–4^
15	3.18 × 10^2^	3.60 × 10^2^	6.60 × 10^–4^
16	3.18 × 10^2^	4.00 × 10^2^	7.66 × 10^–4^
17	3.28 × 10^2^	1.20 × 10^2^	5.34 × 10^–5^
18	3.28 × 10^2^	1.60 × 10^2^	1.28 × 10^–4^
19	3.28 × 10^2^	2.00 × 10^2^	3.02 × 10^–4^
20	3.28 × 10^2^	2.40 × 10^2^	4.14 × 10^–4^
21	3.28 × 10^2^	2.80 × 10^2^	5.82 × 10^–4^
22	3.28 × 10^2^	3.20 × 10^2^	7.87 × 10^–4^
23	3.28 × 10^2^	3.60 × 10^2^	8.51 × 10^–4^
24	3.28 × 10^2^	4.00 × 10^2^	1.03 × 10^–3^
25	3.38 × 10^2^	1.20 × 10^2^	3.31 × 10^–5^
26	3.38 × 10^2^	1.60 × 10^2^	9.09 × 10^–5^
27	3.38 × 10^2^	2.00 × 10^2^	2.98 × 10^–4^
28	3.38 × 10^2^	2.40 × 10^2^	4.81 × 10^–4^
29	3.38 × 10^2^	2.80 × 10^2^	6.77 × 10^–4^
30	3.38 × 10^2^	3.20 × 10^2^	8.89 × 10^–4^
31	3.38 × 10^2^	3.60 × 10^2^	1.08 × 10^–3^
32	3.38 × 10^2^	4.00 × 10^2^	1.24 × 10^–3^

## Methodology

### Random forest and extra tree

The random forest ensemble learning model is a tree-based technique that, like other ensemble learning methods, which is used to enhance the effectiveness of multiple base tree learners^29^. There will then be an unpruned regression tree built for every bootstrapped sample. This is what will happen next. Instead of using all the current predictors, a specified number of *K* base models are picked randomly to perform the function of split possibilities in this stage. This two-step operation will be iterated unto *C* decision trees with the above-mentioned characteristics are optimized, at which point unobserved data can be predicted by gathering the estimations of these *C* trees. Random forest uses a bagging strategy to boost tree diversity via constructing DTs using different training subsets, minimizing the model's total variance^17^. An RF regression predictor is expressed in the following equation:

1 $$\hat{f}_{RF}^{C} \left( x \right) = \frac{1}{C}\mathop \sum \limits_{i = 1}^{C} T_{i} \left( x \right)$$

According to the previous equation, *C* refers to the count of decision trees, x identifies the data point, and *T*_*i*_(*x*) refers to a unique DT built from bootstrap samples and a subset of entry variables. RF can predict out-of-bag error for the time being logging natively using samples which have not been selected in connection with the drive of this shaft during the bagging process. To compute an unbiased prediction of distribution error, this particular sub-association does not make use of any external data^19,30^. Assign substantial scores to each input variable. RF modifies one input variable while holding the others constant, and the model's average decrease is also assigned^19^.

Extra Trees (ET) are an overall tree-based approach like random forest. It strongly randomize both the cut point decision and the particularities of a tree node during its division Extra Tree becomes possible to categorize and regression tasks^31,32^.

As far as the differences are concerned, the two models are identical in that they develop multiple trees and divide nodes applying random subsets of functions, nevertheless, there are two major separations exist: Rather than using optimum splits, the ET uses randomized splits instead of bootstrap observations^33^.

### Gradient boosting

Boosting is also an ensemble learning technique. Boosting comprises a sequence of base predictors rather than a single predictor to average them all together to improve prediction accuracy. In a stage-wise process, base estimators (decision trees here) are successively fitted to eliminate bias. At each phase, a new learner is introduced to optimize the loss function. The first learner reduces the loss function to the smallest possible value using training data^24,34,35^. The residuals from the previous estimators are used by the following estimators. The gradient boosting method steps are depicted in the following Algorithm^24,35,36^:
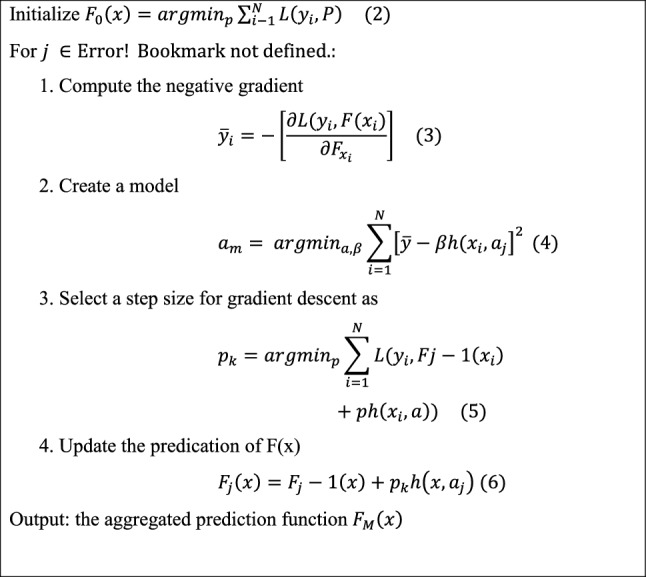


## Results

**Figure 3:**
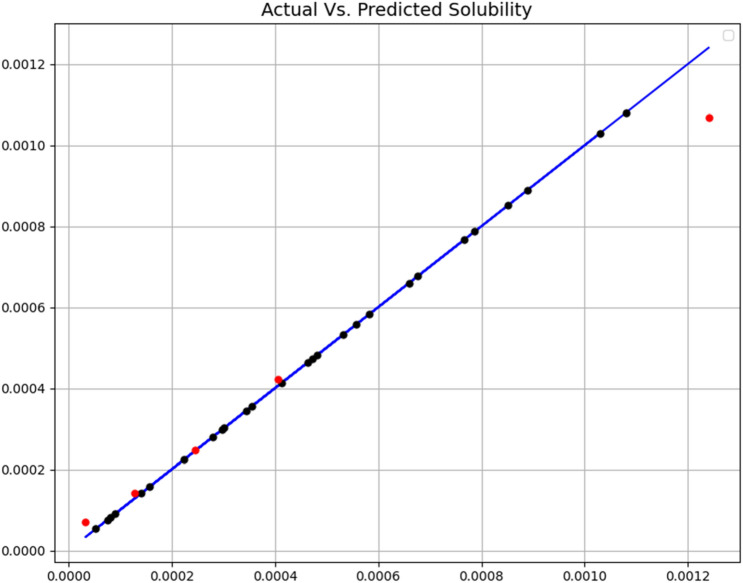
Expected and predicated solubility (ET model).

**Figure 4:**
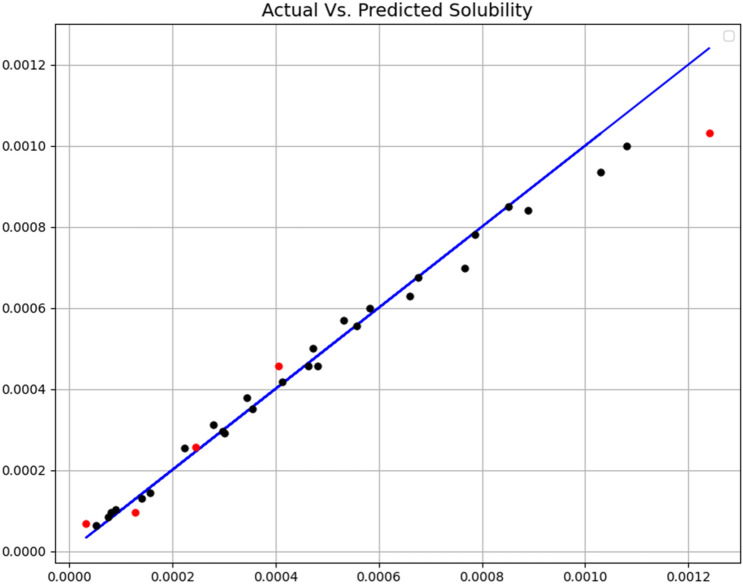
Expected and predicated solubility (RF model).

**Figure 5:**
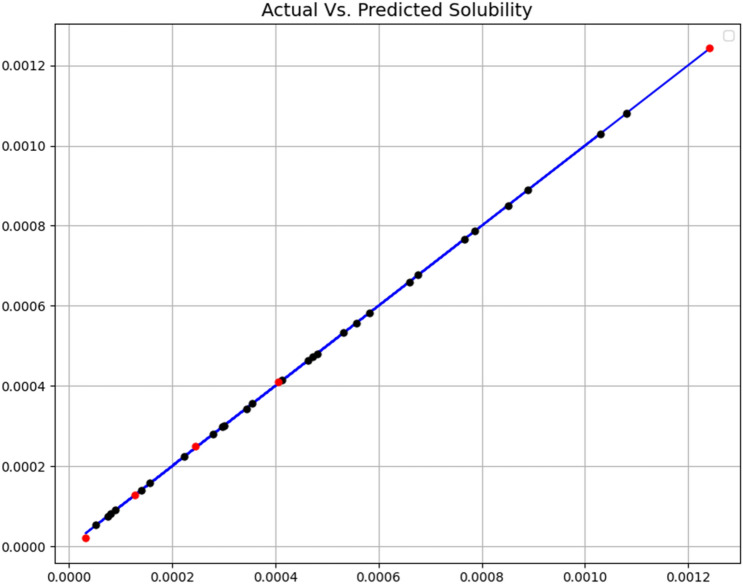
Expected and predicated solubility (GB model).

The tuning of the hyper-parameters of the mentioned models is based on a search grid. All three final models were evaluated by R-square and MSE criteria. Additionally, some visualization results were made, which will be discussed later. Figures 3, 4 and 5 show a comparison of expected values and predicted amounts. In the below figures, the blue line indicates the expected amounts and the points of the predicted values (red for the test data and black for the training data). In addition, Table 2 shows quantitative metrics to compare the three implemented models with the optimal hyper-parameters. Comparison of tabulated results in Table 2 has confirmed the fact that the GB is the most accurate and general model (R^2^ = 0.999 and MSE = 3.78E−11), which has been used as the main model for the rest of the analysis.

**Table 2:** Final model results.

Models	MSE	R^2^
ET	6.29 × 10^–9^	0.999
RF	9.71 × 10^–9^	0.984
GB	3.78 × 10^–11^	0.999

**Figure 6:**
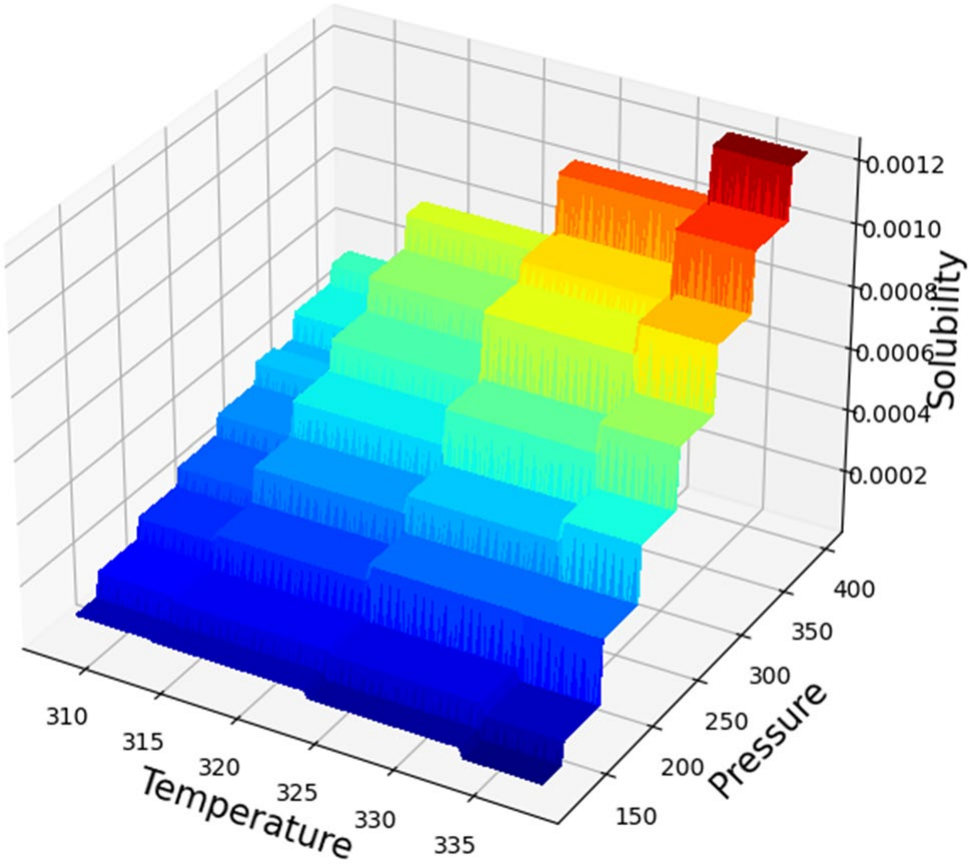
Input–output projection (GB).

**Figure 7:**
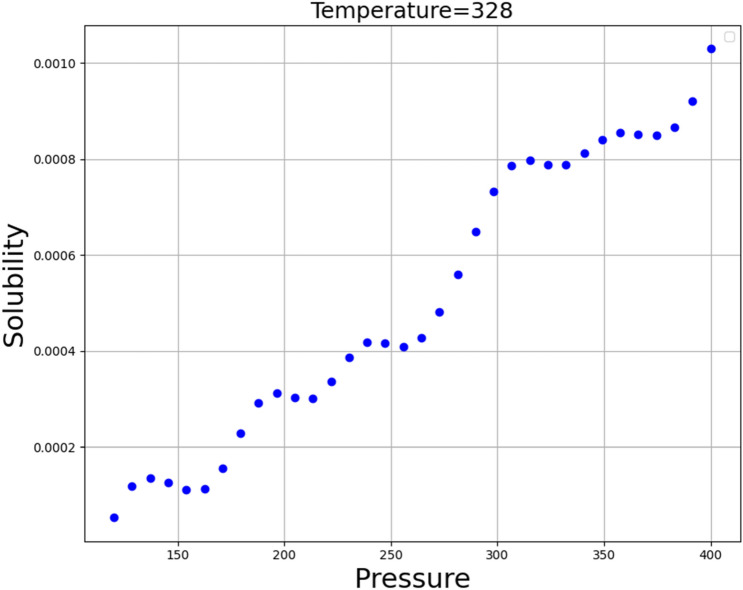
Solubility (mole fraction) based on pressure (bar), temperature (°K).

**Figure 8:**
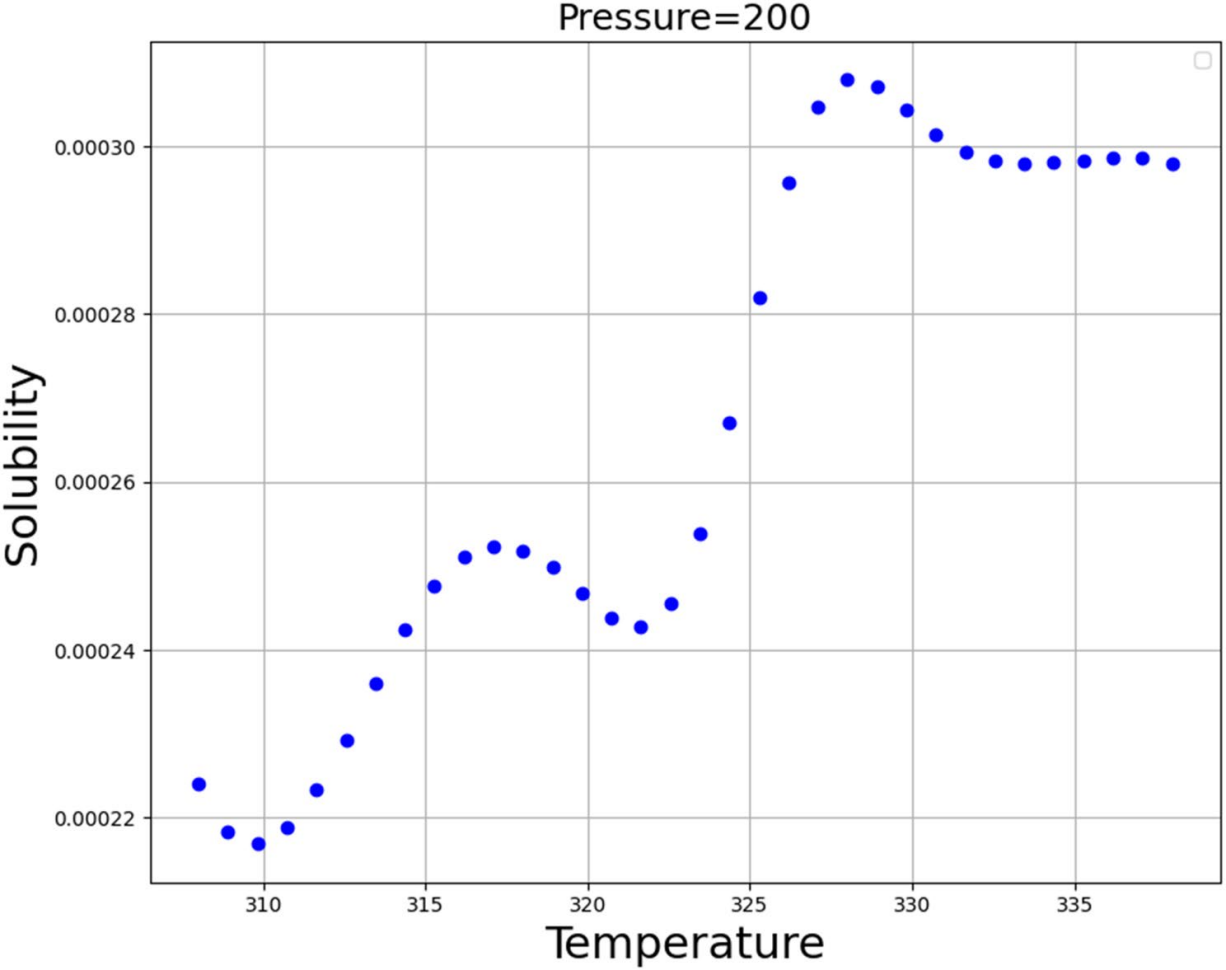
Solubility (mole fraction) base on temperature (°K), pressure (bar).

The simultaneous impacts of temperature and pressure as two prominent input parameters on the solubility as the only output is shown in 3D in Fig. 6. Furthermore, by holding each of the inputs fixed, the two-dimensional Figs. 7 and 8 are displayed. These figures correspond to the reality of the optimal values in Table 3. It can be perceived from the figures that the pressure of system has positive impact on the solubility of Oxaprozin in supercritical system. Indeed, increase in the pressure can improve the solvent density, which consequently intensifies the solvating power of the SC-CO_2_ system. Although pressure has direct connection with the solubility of drug, the impact of temperature is entirely indirect. To evaluate the effect of temperature on drug solubility, the role of sublimation pressure and density above and below the cross-over pressure (COP) must be analyzed. At the pressures above the COP, the encouraging influence of sublimation pressure on solubility dominates the deteriorative impact of density reduction. Therefore, at these pressures, temperature increment significantly enhances the solubility in SC-CO_2_ system. At pressures below the COP, the destructive impact of density decrement overcomes the positive effect of sublimation pressure. Therefore, at these amounts of pressures, increasing the temperature significantly reduces the solubility in SC-CO_2_. By concentrating on Table 3, it is recognized that the pressure and the temperature of 380.4 bar and 333.15 K are the optimum factors for reaching the greatest amount of Oxaprozin solubility.

**Table 3:** Optimal values.

Temperature (K)	Pressure (bar)	Solubility (mole fraction)
333.15	380.4	0.001242

## Conclusion

Now a days, numerous efforts have been made to develop green and efficient solvents to overcome the functional/operational detriments of organic solvents. Nowadays, SC-CO_2_ has been introduced as a prevalently employed liquid solvent to fractionate the valuable components and increase the solubility of drugs in pharmaceutical processes because of its remarkable advantages (i.e., abundancy, cost-effectives, and environmentally benign characteristic). In this paper, disparate types of numerical models were proposed via AI technique to anticipate the optimum value of Oxaprozin in SC-CO_2_. In this study, three ensemble decision tree-based models were used to model the problem: extremely random tree (ET), random forest (RF), and Gradient Tree Boosting (GB). This problem's available data consists of 32 data vectors with two inputs of temperature and pressure and an output of solubility. ET, RF, and GB had MSE error rates of 6.29E−09, 9.71E−09, and 3.78E−11. They also have R-squared scores of 0.999, 0.984, and 0.999, respectively. The final model chosen is GB, with the following optimal values: T = 33.15, *P* = 380.4, and solubility = 0.001242, which shows the greatest amount of Oxaprozin solubility.

## Data Availability

All data are available within the published paper.

## Abbreviations

SC-CO_2_: Supercritical carbon dioxide fluid
AI: Artificial intelligence
NSAID: Nonsteroidal anti-inflammatory
RF: Random forest
GB: Gradient boosting
ET: Extremely random trees
ML: Machine learning
API: Active pharmaceutical ingredient
SCFs: Supercritical fluids
DT: Decision tree
COP: Cross-over pressure
